# Perceptions of Exposure and Mask Use in Wildland Firefighters

**DOI:** 10.3390/toxics12080576

**Published:** 2024-08-07

**Authors:** Tanis Zadunayski, Natasha Broznitsky, Drew Lichty, Nicola Cherry

**Affiliations:** 1 Division of Preventive Medicine, University of Alberta, Edmonton, AB T6G 2T4, Canada; 2 BC Wildfire Service, Ministry of Forests, Victoria, BC V8T 5J9, Canada; 3 Canada Wildfire, University of Alberta, Edmonton, AB T6G 2H1, Canada

**Keywords:** wildland firefighters, smoke perception, respiratory protection, masks, exposures

## Abstract

Wildland firefighters are exposed to airborne particulates, polycyclic aromatic hydrocarbons (PAHs), and other hazardous substances. Respiratory protection is indicated, but information is lacking on the tasks and conditions for which mask wearing should be advised. Studies to assess respiratory protection in wildland firefighters were carried out in western Canada in 2021 and 2023. Sampling pumps measured airborne exposures and urinary 1-hydroxypyrene (1-HP) was assayed to indicate PAH absorption. Participants in 2021 reported the time for which they wore the mask during each task. In 2023, the use of masks was reported, and firefighters rated the smoke intensity. In 2021, 72 firefighters were monitored over 164 shifts and, in 2023, 89 firefighters were monitored for 263 shifts. In 2021, mask wearing was highest for those engaged in initial attack and hot spotting. Urinary 1-HP at the end of rotation was highest for those reporting initial attack, working on a prescribed fire and mop-up. In 2023, firefighter ratings of smoke intensity were strongly associated with measured particulate mass and with urinary 1-HP, but masks were not worn more often when there was higher smoke intensity. The data from the literature did not provide a clear indication of high-exposure tasks. Better task/exposure information is needed for firefighters to make informed decisions about mask wearing.

## 1. Introduction

Seasonal wildland firefighters are exposed to particulate matter from smoke that may result in reduced lung function over the shift [1,2,3,4] and fire season [5,6,7], leading to an increase in respiratory symptoms [8,9], an increase in sputum granulocytes, signifying the inflammation of the respiratory tract [10] and chronic obstructive pulmonary disease (COPD) and pneumonia [11]. Smoke from burning biomass also contains polycyclic aromatic hydrocarbons (PAHs) that may relate to firefighters’ cancer risk [12]. Nevertheless, wildland firefighters traditionally do not wear any respiratory protection, and even when this is made available, its use is intermittent [13,14,15].

In a previous study during 2021, we found that both respiratory and skin exposures to PAHs contributed to end-of-shift urinary 1-hydroxypyrene (1-HP) concentrations, reflecting PAH absorption, and that urinary 1-HP at the end of shift was lower in those randomized to wear an N-95 mask [14]. In our 2023 study, we replicated the relation between respiratory and skin PAH exposures and absorption but found that masks were only protective in conditions with high exposure to particulates in the breathing zone [15].

This report investigates the circumstances under which wildland firefighters elect to wear a mask when one is made available. It assesses the accuracy of firefighters’ perceptions of exposure to airborne particles (as conducted previously by Reinhardt and Ottmar [16] and Navarro et al. [17]) and considers the extent to which this perception influences mask wearing behaviour. It brings together data from fieldwork carried out in Alberta and British Columbia (BC) in 2021 and from the published literature to identify wildland firefighters’ tasks that carry a risk of high exposures. The overall objective was to provide evidence that may be helpful in training wildland firefighters to make informed decisions about when to wear a mask.

## 2. Methods

During the second half of the 2021 fire season (beginning in July 2021, when the COVID-19 pandemic had receded sufficiently to permit field research), wildland fire crews were recruited in Alberta (N = 46) and BC (N = 34) and were followed for 184 firefighter*rotations; of these, 49 were for firefighters randomly allocated an N95 mask. In Alberta, the crews were either helitack (arriving by air to carry out an initial attack on a new fire) or unit crews (carrying out sustained action against a fire). In BC, all were with a unit crew. More details are given in Cherry et al. [14]. All firefighters were asked to complete an end-of-rotation questionnaire with a checklist to record the tasks that they had performed during the rotation and, if allocated a mask, to show on a visual analogue scale how much of the time (from always to never) they wore the mask while carrying out that task. The checklist included 10 firefighting tasks: initial attack, sustained action, prescribed fire, hazard reduction, hot spotting, mop-up, burnout, patrol, gridding, and driving. Initial attack involves action taken to stop the spread of a fire by the first firefighting personnel on scene, whereas sustained action involves fire suppression on a wildfire for an extended time period. A prescribed fire is a pre-planned application of a fire to a specific area as a form of land management. Hazard reduction involves methods to reduce the hazard of a future wildfire, such as controlled burns and slashing. Hot spotting involves checking the spread of a fire and may include cold trailing during mop-up, which involves extinguishing any burning or smouldering material after a fire has been brought under control using water or hand tools. Burnout includes methods used during a planned ignition or prescribed fire. Patrol entails inspecting a section of a control line or perimeter to prevent fire escape after it has been contained, while gridding involves walking through an area looking for hot spots. Driving does not directly manage a fire.

The firefighters also recorded, at the end of rotation, how many days it was since they had been engaged in fighting a fire and gave a spot urine sample that was analyzed for 1-hydroxypyrene (1-HP), a urinary biomarker of absorption of PAHs recommended for use in occupational studies [18]. The end-of-shift urine samples were collected as a spot mid-flow sample into a sterile 100 mL plastic container. These were transported to the Alberta Toxicology Centre in Calgary. They were analyzed for 1-HP, following the liquid chromatography–tandem mass spectroscopy method previously validated [19,20]. Details of the method were given in an earlier publication [19].

During the 2023 fire season, six wildland crews were recruited in BC and followed for three successive days between the end of July and mid-September. Within each crew, firefighters were randomly allocated to one of four conditions: no mask (usual practice) or to wear, as they judged appropriate, one of three types of mask, which were X, a half-face respirator with P100/Multi gas cartridge; Y, a cloth with alpaca filter; and Z, a mesh fabric with carbon filter (Supplementary Materials S1, Figure S1). During each shift, four of the crew carried sampling pumps to measure the total particulate mass and to collect samples for the analysis of PAHs on particles and in the vapour phase, as had been performed in our earlier study [14]. Total particulates were measured from the mixed cellulose ester (MCE) filter used to collect the particulate fraction by gravimetric analysis following NIOSH method 0500 for total particulates. As before, 27 PAHs were assessed on particles and in the vapour phase and subjected to a principal component analysis to derive an overall indicator of PAH exposure with mean = 0 and SD = 1 [14,15]. At the end of each day, firefighters rated their perception of smoke exposure during the day (Supplementary Materials S2, Question D), using visual aids (photographs of firefighters working in smoke) developed and validated against measured particulates by Reinhardt and Ottmar [16]. The photographs showed smoke densities considered by Reinhardt and Ottmar to depict light, medium, heavy, and very heavy levels. Firefighters in the 2023 study were asked to choose the photograph that best matched their perception of typical exposure during that shift and the photograph that best matched the worst exposure. They were also given the option of no smoke exposure. Those allocated a mask were asked if they had worn it at all during that day and, if so, for how many hours they had not worn it. The length of the firefighting day was known from the start and end time for the sampling pumps, which allowed the time worn to be calculated. The firefighters also gave a start- and end-of-shift spot urine sample for the estimation of 1-HP.

In both 2021 and 2023, the analysis used the natural logarithm (to reduce skew) of 1-HP corrected for creatinine as a biomarker of exposure, excluding samples with creatinine <30 mg/dL or >300 mg/dL [21,22]. In 2021, the contribution of tasks to PAH exposure was estimated from end-of-rotation urinary 1-HP. Data on task exposures were also extracted from the literature after a review of all articles in PubMed identified through a search for ‘wildland firefighter’ and with follow-up searching of any additional references listed in the papers extracted. Task exposure data extracted from the literature, supplemented by the fieldwork data from 2021, were used to identify and assess high-exposure tasks.

The schema for data collection and analysis is summarized in Table 1. Data from 2021 were used to examine two questions: (1) which tasks were associated with higher mask use (interpreted here as indicating higher perceived risk from airborne exposure) and (2) which tasks were associated with higher excretion of urinary 1-hydroxypyrene (indicating higher PAH absorption by any route). Data from 2023 (in which task information was not collected) were used only to assess the relation between the cross-shift perception of smoke density and reported mask wearing. To simplify the presentation, the data from 2023 are considered first.

### Statistical Methods

As the same firefighter contributed data on multiple days/rotations, all analyses used multilevel modelling to allow for clustering within each firefighter. The 2023 ratings for perceived smoke density, regrouped to three categories (none or low (none or light smoke levels), moderate (medium levels), high or very high (heavy or very heavy levels)) of typical and worst conditions, were related in a linear mixed effects model to measured particulate exposure (as the mean of the estimates from the four pumps for that crew on that day), estimated PAH exposure, and post-shift log urinary 1-HP/creatinine ng/g. An analysis stratified by rotation number (1st, 2nd, 3rd) assessed the contribution of perceived smoke density to log reported hours of mask wearing during each shift. To explore factors associated with the extent of mask use (time worn) in those allocated a mask in 2021, a mean rating of time worn (from never (0) to always (95)) was calculated for each firefighter, using the ratings from each task that they reported. This personal mean score for each rotation was then related, in multivariable regression analyses, to the tasks reported and potential confounding factors (age, gender, unit type, and rotation number) to determine the contribution of each task to the overall pattern of use. The relation of 1-HP at the end of rotation in 2021 to tasks reported for that rotation was determined in a multilevel multivariable regression, allowing for the recency of firefighting activity (same day or earlier) and, in a model restricted to those allocated a mask, to tasks adjusted for mask wearing. The analysis was carried out in Stata 18 (StataCorp. 2023, *Stata Statistical Software: Release 18*, StataCorp LLC, College Station, TX, USA) and used a two-sided probability of <0.10 as indicating a difference in possible interest.

## 3. Results

In 2023, 89 firefighters were monitored for 263 shifts*firefighter observations. Overall, firefighters reported wearing their masks for 30% of their shift (mean length 10.7 h, range 6.6–13.3). Each BC firefighter rated their perceptions of the typical and worst smoke density during the monitored shift. Table 2 shows the mean particulate mass, the mean of the indicator of PAH exposure, and, for those with acceptable creatinine levels, the mean urinary 1-HP/creatinine. There was a clear and significant trend between subjective ratings of smoke density and objective estimates of exposure. No equivalent relation was seen with mask wearing (Table 3) for the 66 firefighters allocated masks in 2023. Those who recorded a perception of high ‘typical’ or ‘worst’ exposure did not report wearing their masks for longer than those who recorded little or no smoke: there was no significant trend between log hours of mask wearing and recorded perception of smoke density on the first, second, or third monitored fire day or overall.

In 2021, 73 firefighters completed an end-of-rotation questionnaire for a total of 155 rotations with data on tasks carried out during the rotation (Table 4). Three quarters of firefighters reported mop-up and driving, with only a third reporting initial attack, and rather few (14%) working on a prescribed fire. Table 4 also shows the mean time rating for mask wearing in each task by a firefighter allocated an N95 mask. The highest use of masks was during initial attack, sustained action, and hot spotting, with driving and patrolling having the least use. The next column shows, for each task, the deviation from the firefighter’s overall rating of time used. On this ipsative scale, working on a prescribed burn is seen to have the highest time use of a mask. The final column shows how each task reported contributed to the overall mean mask use: only initial attack and hot spotting were significantly related to an increase in mask use. Using the overall scale as a measure, mask use was unrelated to gender or crew type (unit crew or helitack), with a tendency toward greater use in those aged 30 years or older. Use decreased with successive rotations, up to the third rotation. Three firefighters in one BC crew were monitored for a fourth rotation, where the reported use was high (Supplementary Materials S3, Table S1).

There were 136 rotations*firefighters with estimated 1-HP and completion of the end-of-rotation questionnaire in 2021. Very few firefighters smoked cigarettes or ate charbroiled food 24 h prior to giving a urine sample. The relation between reported tasks and end-of-rotation log urinary 1-HP/creatinine is shown in Table 5. Those whose urinary sample had been collected at the end of a day of active firefighting had higher 1-HP than those occupied in other ways on that day. Multivariable regression suggested (model 1) that mop-up and working on a prescribed fire were more associated (but only marginally) with higher PAH absorption (indicating higher exposure), and gridding was associated with lower PAH absorption. In those allocated a mask (models 2 and 3), the contribution of tasks was more evident, with initial attack associated with higher 1-HP and hazard reduction, and gridding associated with lower absorption. When the extent of personal mask wearing was included (model 3), initial attack, sustained action, and working on a prescribed fire were associated with higher 1-HP, while burnout, patrolling, and driving were associated with lower 1-HP. In this model, adjusting for tasks, those reporting greater use of their masks had lower 1-HP.

Table 6 summarizes the measured particulate exposure for different tasks as reported in the literature. There are data from four groups: Reinhardt and Ottmar [16,23], Gaughan et al. [2], Adetona (including Wu) [24,25,26], and Navarro et al. [17]. The tasks of ‘holding’ and ‘mop-up’ are reported by three of the four groups, and a number of other tasks are reported by two. There is variation in the particulate fractions measured (PM2.5, 3.5, 4.0), in the way the task was defined or measured, and in the type of fire (prescribed or wildfire), resulting in a range of estimates for tasks reported more than once. Table 7 reports cross-shift exposures from the literature [2,17,23,24,25,26,27,28,29,30,31,32,33,34,35]. The current study only collected (in 2023) the total particulate mass (with the great majority expected to be fine or ultrafine [36]), similar to that reported for full shifts by Gaughan et al. [2], Kelly [31], Reinhardt and Broyles [34], and Navarro [17].

## 4. Discussion

Firefighters allocated masks reported a pattern of wearing that differed between tasks (with masks most worn for initial attack, during hot spotting, and during sustained action). Perceptions of smoke density were found to be related to objective measures of exposure but did not appear to influence mask usage. From the task data in the 2021 fieldwork, high exposures (reflected in urinary 1-HP) were found for initial attack, sustained action, and working on a prescribed fire. Data from the literature, from direct measurement of particulate exposure for specific tasks, were insufficient to warrant strong recommendations regarding tasks with a risk of high exposure, but were largely consistent with the observations from the current study that high-exposure tasks may include initial (direct) attack and prescribed fires. None of the shift values for wildfires in Table 7 exceeded the suggested Occupational Exposure Limit (OEL) of 0.7 mg/m^3^ [28], although some prescribed burns were reported to do so, together with half the estimated values for holding and mop-up, as summarized in Table 6. The long-term respiratory ill health found in wildland firefighters [11] would suggest that an exposure limit of 0.7 mg/m^3^, although seldom exceeded, is too low to protect firefighters from chronic ill health.

While existing studies were carried out with evident concern for protocol and scientific rigour, the measurement of specific tasks is demanding and will always be a compromise taking account of the realities on the ground: the demands of the job change with fire behaviour. Short of a one-on-one pairing between an occupational hygienist and an individual firefighter with real-time measurement of particulates and video recording of tasks, attempts to use field data to identify high-risk tasks may not be successful. In reviewing the literature, we had hoped to identify tasks that were indisputably at risk to use as a starting point for firefighter education on mask use, but the results are too few and too varied to provide a secure basis for such training.

The fieldwork data reported here have limitations. Detailed task information was collected only in 2021 and related to tasks carried out during the whole rotation. Reporting on tasks on a single day, with urinary 1-HP at the start and end of that day, together with measurements of breathing zone contaminants generated by the task, would have given more precise information. The relation between mask wearing and tasks was recorded in 2021 for only a small number of firefighters, all of whom were allocated an N95 mask. The preference for wearing a mask for initial attack, for example, might differ importantly with the type of mask and the firefighter’s perception of the protection afforded [15]. Further, mask wearing was part of a structured investigation in which those allocated masks were encouraged to wear them as much as possible. Outside the research environment, firefighters might have been a great deal more discriminating in their mask use. A further limitation is that the perception of risk and mask wearing considered here relates only to particulates and substances (including PAHs) that they may contain. Firefighters are also exposed to breathing zone gasses and vapours that are not mitigated by particulate masks. Even when worn optimally, masks do nothing to reduce exposures by skin absorption, which, for PAHs, may be more important than absorption through the lungs in some circumstances [15]. The allocation of firefighters to mask wearing in Alberta in 2021 was randomized by crew. Among the seven crews, two were allocated to mask wearing, with neither of these two being a unit crew; as such, the tasks recorded by those wearing masks differed from the overall group of firefighters, with more of those allocated a mask reporting initial attack.

The failure to find any relation between perceived smoke density and mask wearing was disappointing, as density ranking has the potential to be developed as a user-friendly tool to help a firefighter make an informed decision about when to wear a mask. A personal monitor for particulates has been suggested [37] to indicate to wildland firefighters when some control is needed, but we are not aware that such a monitor has been developed. Our attempt to pull together objective data on the tasks with the highest exposures was undertaken in the hope that it might provide a framework for decision making in terms of mask use, with task data complemented by clear instruction on the interpretation of heavily contaminated air.

We do not know if there is a safe level of particulate exposure for wildland firefighters, but wearing a mask may interfere with communication and respiration and cause discomfort, and it may be unrealistic and unnecessary to wear them for tasks that are not identified as exposure. If the decision is to be left to the firefighter, then they need credible evidence as to the tasks and circumstances that carry a risk of high exposure. A well-funded programme of work to complement and extend that reported by Navarro et al. [17] would be a major contribution to wildland fighter wellbeing. We now know [11] that repeated exposures result in long-term damage to wildland firefighters’ respiratory health, and investment in the best possible materials to inform choices seems to be a high priority to mitigate risk. In 2023, the highest measured exposure was on a day with no direct exposure to burning fires but with an inversion causing the valley to be filled with wildfire smoke. The firefighters in that crew rated the density as very high. No checklist of tasks, however valid, would have trumped their own perception regarding smoke intensity. Any training on intelligent mask wearing must seek to blend knowledge of high-exposure tasks with action based on the assessment of local circumstances.

## Figures and Tables

**Table 1:** Summary of data used from each year of fieldwork.

	Year in Which Data Were Collected	
	2021	2023
Participants	Unit crew and helitack	Unit crews
Province	Alberta and British Columbia	British Columbia
Number of Participants	80	89
Number of Rotations	184	263
Task Data ^1^	All	None
Mask allocation	N95 = 49	One of three types ^2^ = 66
Mask use information	Time for each task	Time over each shift
Rating of smoke density	None	For each shift
Outcome measure	Urinary 1-HP	Hours of mask use

^1^ Report of performing up to 10 firefighting tasks during the rotation and time spent wearing an allocated mask during each task. ^2^ See Supplementary Materials S1.

**Table 2:** Relation between perceived typical and worst smoke density ratings by particulate mass (mg), estimated PAH exposure, and urinary 1-HP/creat (ng/g).

	Typical Exposure Rating						Worst Exposure Rating					
	Particulate Mass (mg)											
Firefighter Smoke Exposure Rating	Mean	SD	β *	95% CI	*p*	N	Mean	SD	β *	95% CI	*p*	N
Low	0.55	0.27	0	–	–	171	0.54	0.26	0	–	–	104
Moderate	0.78	0.35	0.18	0.11 to 0.26	<0.001	71	0.59	0.25	−0.02	−0.09 to 0.06	0.652	90
High	0.88	0.33	0.28	0.16 to 0.39	<0.001	21	0.86	0.39	0.23	0.15 to 0.31	<0.001	68
	**PAH Indicator**											
Low	−0.27	0.66	0	–	–	171	−0.35	0.58	0	–	–	104
Moderate	0.64	0.89	0.59	0.44 to 0.73	<0.001	71	0.10	0.92	0.06	−0.09 to 0.22	0.431	90
High	0.90	0.87	0.77	0.53 to 1.00	<0.001	21	0.68	0.86	0.55	0.37 to 0.72	<0.001	68
	Log Urinary 1-HP (ng/g) **											
Low	5.64	0.80	0	–	–	137	5.48	0.81	0	–	–	84
Moderate	6.03	0.87	0.16	−0.01 to 0.33	0.070	59	5.82	0.75	0.20	0.03 to 0.38	0.024	78
High	6.07	0.75	0.24	−0.06 to 0.53	0.114	18	6.23	0.78	0.42	0.21 to 0.64	<0.001	52

* From a linear mixed effects model allowing for repeat rotations by the same firefighter. ** Present only for those with creatinine 30–300 mg/dL.

**Table 3:** Relation of log mean hours wearing a mask with smoke density ratings by fire day.

		Fire Day 1			Fire Day 2			Fire Day 3			Overall		
Typical Smoke Density		Mean	SD	N	Mean	SD	N	Mean	SD	N	Mean	SD	N ^2^
Log Hours	Low	1.59	0.83	38	1.04	1.23	39	0.86	1.11	48	1.14	1.11	125
Worn	Moderate	1.54	0.80	23	1.10	1.16	23	1.48	0.95	10	1.35	0.99	56
	High	1.45	1.02	8	1.99	0.10	2	0.67	0.87	6	1.22	0.98	16
	Overall	1.55	0.83	69 ^1^	1.09	1.19	64	0.94	1.08	64	1.21	1.07	197
	p (linearity)	0.676			0.468			0.702			–		
Worst Smoke Density													
Log Hours	Low	1.53	0.87	15	1.24	1.17	25	1.01	1.12	29	1.20	1.10	69
Worn	Moderate	1.85	0.58	24	0.95	1.21	28	0.84	1.11	20	1.22	1.09	72
	High	1.33	0.93	30	1.12	1.23	11	0.96	1.02	15	1.19	1.01	56
	Overall	1.56	0.83	69 ^1^	1.09	1.19	64	0.94	1.08	64	1.21	1.07	197
	p (linearity)	0.232			0.625			0.818			–		
Multivariable, Multilevel Regression Adjusting for Fire Day													
Typical Smoke Density		β	95% CI		*p*=								
Log Hours	Low	0.00	–		–								
Worn	Moderate	0.23	−0.09 to 0.56		0.164								
	High	0.24	−0.18 to 0.67		0.262								
Worst Smoke Density													
Log Hours	Low	0.00	–		–								
Worn	Moderate	0.03	−0.25 to 0.30		0.840								
	High	0.26	−0.07 to 0.58		0.121								

^1^ Three firefighters had an extra fire day; ^2^ firefighters*fire day.

**Table 4:** Reported tasks by time spent wearing an allocated mask as recorded and as deviation from firefighters’ overall time rating.

	Number Reporting Each Task				Visual Analogue Rating of Time Wearing a Mask						
	Whole Sample ^1^		Allocated a Mask		As Recorded		As Deviation from Own Mean		Multivariate Model ^2^		
Task	N	%	N	%	Mean	SD	Mean	SD	β	95% CI	*p*
Initial Attack	52	33.5	23	54.8	55.0	33.1	7.1	14.6	13.57	1.15 to 25.99	0.032
Sustained Action	107	69.0	31	73.8	48.0	32.4	7.6	16.4	−11.56	−30.33 to 7.22	0.228
Prescribed Fire	22	14.2	11	26.2	39.7	36.4	12.2	24.2	−11.05	−56.72 to 34.62	0.635
Hazard Reduction	32	20.6	12	28.6	23.5	26.6	−6.7	24.9	−0.58	−35.85 to 34.70	0.974
Hot Spotting	105	67.7	33	78.6	45.8	33.1	4.9	11.9	29.93	7.10 to 52.78	0.010
Mop-Up	119	76.8	35	83.3	44.4	32.9	3.9	16.4	−12.83	−33.67 to 8.01	0.228
Burnout	30	19.4	12	28.6	36.9	29.4	6.9	24.6	7.50	−20.93 to 35.94	0.605
Patrol	93	60.0	21	50.0	14.9	26.3	−16.8	20.1	−8.49	−25.09 to 8.12	0.317
Gridding	72	46.5	22	52.4	35.7	38.3	−5.3	18.2	8.83	−4.08 to 21.75	0.180
Driving	115	74.2	23	54.8	10.3	20.3	−22.2	26.0	−10.95	−25.92 to 4.03	0.152
N: Observations	155	100.0	42	100.0	42		42		42		
N: Firefighters	69		18		18		18		18		

^1^ Firefighters who completed questionnaire, regardless of assigned practice (no mask or mask). ^2^ Linear mixed effects model to assess the relation of carrying out a task to mean overall time wearing a mask.

**Table 5:** Linear mixed effects model to assess the effect of the task on end-of-rotation log 1-hydroxypyrene/creatinine (ng/g) with adjustment for fire exposure on the day that the urine sample was collected.

	All Firefighters (Model 1)			Those Allocated a Mask (Model 2)			Those Allocated a Mask Adjusted for Mask Wearing (Model 3)		
	β	95% CI	*p* *	β	95% CI	*p* *	β	95% CI	*p* *
Initial Attack	0.00	−0.33 to 0.33	0.997	0.42	−0.05 to 0.89	0.083	0.42	0.12 to 0.73	0.007
Sustained Action	0.20	−0.22 to 0.61	0.348	0.14	−0.47 to 0.76	0.645	0.34	−0.01 to 0.68	0.058
Prescribed Fire	0.50	−0.05 to 1.06	0.076	0.89	−0.30 to 2.07	0.141	0.87	0.21 to 1.52	0.009
Hazard Reduction	−0.01	−0.50 to 0.48	0.977	−0.83	−1.70 to 0.05	0.066	−0.39	−0.87 to 0.08	0.107
Hot Spotting	−0.17	−0.59 to 0.26	0.436	0.01	−0.68 to 0.70	0.982	0.06	−0.33 to 0.45	0.751
Mop-Up	0.42	−0.01 to 0.85	0.054	0.52	−0.28 to 1.33	0.198	0.35	−0.06 to 0.76	0.098
Burnout	0.11	−0.33 to 0.55	0.627	−0.38	−1.21 to 0.46	0.377	−0.49	−0.97 to 0.01	0.045
Patrol/Recon	0.16	−0.22 to 0.53	0.410	−0.28	−0.96 to 0.40	0.420	−0.56	−0.90 to −0.21	0.002
Gridding	−0.46	−0.84 to 0.07	0.020	−0.63	−1.22 to −0.04	0.037	−0.01	−0.35 to 0.33	0.962
Driving	−0.27	−0.70 to 0.16	0.220	−0.46	−1.09 to 0.16	0.148	−0.85	−1.22 to −0.49	<0.001
Firefighting on the Day of the Urine Sample	0.40	0.04 to 0.75	0.027	1.00	0.34 to 1.66	0.003	1.00	0.65 to −1.35	<0.001
Mean Mask Wear	–	–	–	–	–	–	−0.01	−0.02 to −0.01	<0.001
N: Observations	136			34			34		
N: Firefighters	68			18			18		

* From a linear mixed effects model allowing for repeat rotations by the same firefighter.

**Table 6:** Reported respirable particulate matter (RPM) among types of tasks in the current literature (mg/m^3^).

				Study Reference			
Type of Task	Reinhardt and Ottmar		Gaughan et al. ^b^ [2]	Adetona et al. ^b^		Wu et al. ^b^ [26]	Navarro et al. ^a^ [17]
	[16] ^a^	[23] ^b^		[24]	[25]		
Burn/Crew Boss		1.32					
Digging/Constructing Fireline	0.99		0.49				
Direct Attack	0.51	4.04					0.65 ^2^
Engine Operator	0.42	1.37					0.30
Firing							0.43
Gridding	0.50						
Hiking							0.26
Hold and Mop-Up	0.71						
Holding	3.75	1.56		0.63	0.34	1.47	0.37
Holding Boss		1.81					
Indirect Suppression							0.34
Lead Workers ^1^			0.14				
Lighting	0.34	0.75		<0.5	0.24	1.10	
Line Workers			0.33 ^2^				
Mobile Attack	2.49						
Mop-Up	1.84	0.75	0.51				0.34
Other						1.91 ^3^	0.15 ^4^
Sawyer	0.65	2.93	0.52				
Staging							0.20
Swamper	0.67		0.69				

^a^ RPM at wildfires. ^b^ RPM at prescribed fires. ^1^ Oversee crew operations. ^2^ Includes constructing a fireline. ^3^ Other: heli-base operations, gridding the green and gridding the black; both involve looking for hot spots in burned and unburned areas of fire perimeter. ^4^ Other: burn boss, lighting and holding.

**Table 7:** Reported mean ^1^ of respirable particulate matter (RPM) among types of fires in the current literature (mg/m^3^).

		Type of Fire	
Study Reference	Wildfire	Prescribed Fire	Initial Attack Fire
Adetona et al. [27]		0.26	
Adetona et al. [24]		0.53	
Adetona et al. [25]		0.24	
Hejl, et al. [28]		0.65	
Neitzel et al. [29]		1.05 ^a^	
Reisen and Brown [30]		0.60	
Wu et al. [26]		1.43	
Gaughan et al. [2]	0.39		
Kelly [31]	0.37		
Kelly [32]	0.49		
Navarro et al. [33]	0.51		
Navarro et al. [17]	0.32		
Reinhardt and Broyles [34]	0.35	0.32	0.13
Reinhardt and Ottmar [23]	0.50 ^b^	0.63 ^b^	0.02 ^b^
	0.72 ^c^	1.00 ^c^	1.11 ^c^
Reisen et al. [35]	0.42	0.72	

^1^ All studies reported geometric mean, except for Neitzel et al. [29] and Kelly [31,32], who report arithmetic mean. ^a^ Representative of full-shift exposure. ^b^ Shift average time-weighted average (TWA) of paid hours during the shift. ^c^ Fireline average TWA for time at the fire scene.

## Data Availability

Anonymized datasets may be made available in discussion with the corresponding author.

## Supplementary Materials

The following supporting information can be downloaded at: https://www.mdpi.com/article/10.3390/toxics12080576/s1. Figure S1. Masks Allocated in 2023; Questionnaires at start and end of fire day (2023); Table S1. Relation between mean mask use and potential confounders.
