# Supplementary material for: Perceptions of Exposure and Mask Use in Wildland Firefighters
Source: Toxics. 2024 Aug 7;12(8):576. doi: 10.3390/toxics12080576 (PMC11360241; doi:10.3390/toxics12080576)
Supplement: Supplementary file 1 [file toxics-12-00576-s001.zip › toxics-3103602-supplementary.pdf]

toxics MDPI

Perceptions of exposure and mask use in wildland firefighters

Tanis Zadunayski , Natasha Broznitsky, Drew Lichty and Nicola Cherry

Supplementary Materials

1) Supplementary Figures
   Figure S1. Masks Allocated in 2023
2) Questionnaires at start and end of fire day (2023)
3) Supplementary Tables
   Table S1. Relation between mean mask use and potential confounders

Supplemental Materials S1

Figure S1. Masks allocated in 2023.

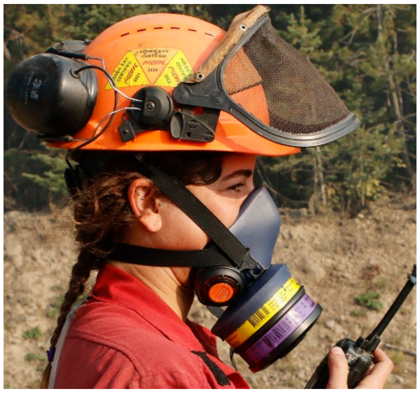	Mask X	- Half face negative pressure respirator<br>- Air purifying respirator<br>- Reusable<br>- Cartridge: P100 multi-gas
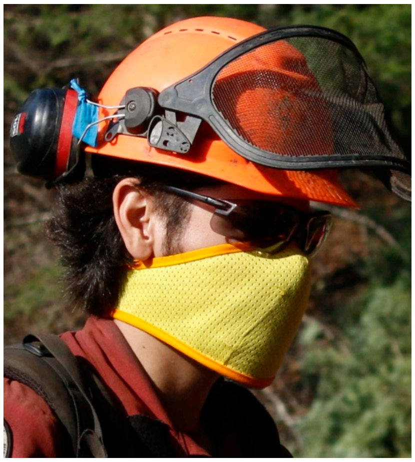	Mask Y	- Fire resistant cloth mask<br>- Reusable<br>- Washable filter<br>- Filter: Alpaca fibre
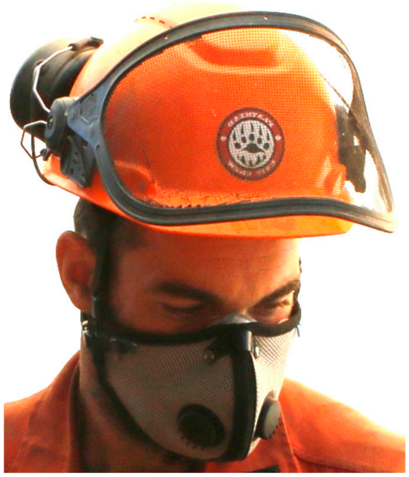	Mask Z	- Air purifying mask<br>- Reusable<br>- Mesh fabric mask<br>- Filter: F3 high airflow+active carbon

Supplemental Materials S2

Questionnaires at Start and End of Fire Day (2023)

The following questionnaires were administered to participants at the start of the fire day shift and at the end of the fire day shift.

Wildland Firefighter Start of Fire Day Questionnaire

A. Symptoms

We are interested in any symptoms you have now (before starting this fire day). Please put an ’X’ on each of the scales below to indicate the <u>most</u> you have been bothered by the symptom since getting up this morning

Sore itchy or running **eyes** — Not at all |———————| Very bothered

Not at all |———————| Very bothered

Sore, itchy or running **nose** — Not at all |———————| Very bothered

Sore or rough-feeling **throat** — Not at all |———————| Very bothered

Coughing

B. **Smoking and other exposures** to polycyclic aromatic hydrocarbons (PAHs)?

B1. When did you last smoke a cigarette, pipe or cigar containing tobacco (do not include vaping or cannabis here)?

☐ In the last 24 hours?

☐ Not in the last 24hrs, but in the last three months

☐ More than 3 months ago

☐ Never smoked as much as 1 cigarette, pipe or cigar a day for as much as a year

B2. .In the last 24 hours have you:

Vaped? ☐ Yes ☐ No

Smoked cannabis? ☐ Yes ☐ No

Chewed tobacco? ☐ Yes ☐ No

Spent time with others who were smoking tobacco? ☐ Yes ☐ No

Spent time around a fire pit or BBQ? ☐ Yes ☐ No

Eaten BBQ or smoked meat or fish? ☐ Yes ☐ No

B3. Before today, how many days ago did you last work on a fire? Count yesterday as one day ago___________________

B4. Before today, for how many days have you worn this Nomex without it being washed? ________

C. Personal details and facial hair

C1 What is your age now? __________years

C2 Do you identify as: ☐ Male

☐ Female (go to D1 below)

☐ Other

If male or other:

C3 Do you now have facial hair (a beard, moustache or sideburns)? Do not include here stubble from not shaving recently ☐ Yes ☐ No (Go to C4)

If yes, please show us on the diagram below which parts of your face have hair then go to D1 below

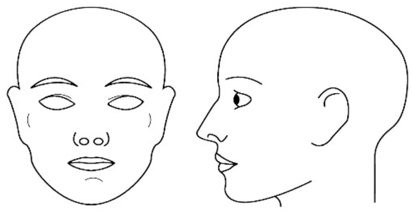

C4: **If no beard, moustache or sideburns**:

Have you shaved your face today? ☐ Yes ☐ No

If no:

How many days ago did you last shave your face?________________(days)

D1 Date today: _____day______month______year

Wildland Firefighter End of Fire Day Questionnaire

A. Symptoms

We are interested in any symptoms you have experienced since starting work today Please put an 'X' on each of the scales below to indicate the most you have been bothered by the symptom during this fire day.

Sore itchy or running **eyes** — Not at all |————————| Very bothered

Not at all |————————| Very bothered

Sore, itchy or running **nose** — Not at all |————————| Very bothered

Sore or rough-feeling **throat** — Not at all |————————| Very bothered

Coughing

B. Exposure to polycyclic aromatic hydrocarbons (PAHs).

Since starting work today have you:

Smoked a cigarette, pipe or cigar containing tobacco? ☐ Yes ☐ No

Chewed tobacco? ☐ Yes ☐ No

Vaped? ☐ Yes ☐ No

Smoked cannabis? ☐ Yes ☐ No

Spent time with others who were smoking tobacco ☐ Yes ☐ No

Eaten BBQ or smoked meat or fish? ☐ Yes ☐ No

C. Use of a mask.

C1. Were you allocated one of the three types of trial masks to wear today? ☐ Yes ☐ No

If yes what sort was it?

☐ Sundstrom SR 100 Half Face Respirator

☐ Fair Air Fire Mask

☐ RZ Mesh Mask

C2. **If not allocated a trial mask:**

Did you wear any type of mask today? ☐ Yes ☐ No (Go to section D1 below)

If yes, was this an N95 ☐ Yes ☐ No ☐ Not sure

If no, what sort of mask was it?___________________________

C3. How often did you wear a mask today?

All day ☐

Most of the day ☐

About half of the day ☐

Less than half the day ☐

Never or almost never ☐

Please estimate the total number of hours you did ***NOT*** wear it while at the fire today_____hrs

C4. If you did **not wear the allocated mask at all**, why did you choose not to?
____

C5. If you wore **a mask at all** today mark an 'X' on the scales below to tell us about your experience while wearing the mask.

Not at all |———————| Very much

Was it comfortable?

Not at all |———————| Very much

Did it fit well?

Not at all |———————| Very much

Did it make breathing difficult?

Not at all |———————| Very much

Did if make your firefighting work more difficult?

Not at all |———————| Very much

Did you feel protected from the smoke/ash?

C6. Do you have any additional comments about wearing the mask?

____

____

____

D. Smoke and Dust Levels while Actively Attacking Burning Fires

D1. How would you rate **typical** smoke and dust levels while you were performing this task?
Please refer to the smoke level information sheet for a description of each level

☐ None ☐ Light ☐ Medium ☐ Heavy ☐ Very Heavy

D2. How would you rate **the worst smoke and dust levels that you experienced** while you were performing this task? *Refer to the smoke level information sheet for a description of each level*

☐ None ☐ Light ☐ Medium ☐ Heavy ☐ Very Heavy

E1. Date today:________day__________month__________year

THANK YOU FOR COMPLETING THIS QUESTIONNAIRE

Supplemental Materials S3

Table S1. Relation between mean mask use[1] and potential confounders.

		Mean	SD	β	95% CI	p
Sex	Female	30.43	40.42	0.00	–	–
	Male	40.86	28.03	10.90	-18 to 40.74	0.474
Age	16-24	27.05	33.92	0.00	–	–
	25-26	47.80	36.39	19.76	-8.06 to 47.58	0.164
	27-29	39.09	16.54	11.29	-17.28 to 39.87	0.439
	30-56	50.71	26.64	25.76	-4.71 to 56.23	0.097
Crew type	Helitack	41.69	35.77	0.00	–	–
	Unit	35.46	23.00	-12.42	-35.25 to 10.41	0.286
Rotation	1	42.87	28.26	0.00	–	–
	2	37.34	33.72	-7.80	-23.56 to 7.95	0.331
	3	22.90	25.16	-19.10	-38.45 to 0.26	0.053
	4	65.92	27.78	16.10	-13.40 to 45.60	0.285
N		42	–	–	–	–

[1] Mean rating (visual analogue scale) of how much time was spent wearing a mask.
